# Porphyrin Diacid-Polyelectrolyte Assemblies: Effective Photocatalysts in Solution

**DOI:** 10.3390/polym8050180

**Published:** 2016-05-04

**Authors:** Sabine Frühbeißer, Giacomo Mariani, Franziska Gröhn

**Affiliations:** 1 Department of Chemistry and Pharmacy, Friedrich-Alexander University Erlangen-Nürnberg, Interdisciplinary Center for Molecular Materials (ICMM), Egerlandstraße 3, Erlangen 91058, Germany; sabine.fruehbeisser@fau.de (S.F.); giacomo.mariani@fau.de (G.M.); 2 Institut Laue-Langevin DS/LSS, 71 Avenue des Martyrs, Grenoble F-38000, France

**Keywords:** self-assembly, porphyrins, supramolecular catalyst, supramolecular structures, polyelectrolytes, photocatalysis

## Abstract

Developing effective and versatile photocatalytic systems is of great potential in solar energy conversion. Here we investigate the formation of supramolecular catalysts by electrostatic self-assembly in aqueous solution: Combining positively charged porphyrins with negatively charged polyelectrolytes leads to nanoscale assemblies where, next to electrostatic interactions, π–π interactions also play an important role. Porphyrin diacid-polyelectrolyte assemblies exhibit a substantially enhanced catalytic activity for the light-driven oxidation of iodide. Aggregates with the hexavalent cationic porphyrin diacids show up to 22 times higher catalytic activity than the corresponding aggregates under neutral conditions. The catalytic activity can be increased by increasing the valency of the porphyrin and by choice of the loading ratio. The structural investigation of the supramolecular catalysts took place via atomic force microscopy and small angle neutron scattering. Hence, a new facile concept for the design of efficient and tunable self-assembled photocatalysts is presented.

## 1. Introduction

Many functional structures in nature are based on non-covalent self-assembly principles. In the last years, self-assembly has emerged as a powerful method to create supramolecular structures of various sizes, shapes, architectures and functionalities [1,2,3,4,5,6,7,8,9,10,11,12]. Due to their size scale and versatile chemistry, polymers are very suitable building blocks to form a variety of stable assemblies in solution. In particular, polyelectrolytes have been successfully used for the formation of a variety of polyelectrolyte complexes, polyelectrolyte-surfactant complexes and in layer-by-layer deposition [13,14,15,16,17,18,19,20,21,22,23,24]. More recently, electrostatic self-assembly of multivalent organic counterions with oppositely charged polyelectrolytes has allowed formation of a broad range of nanoscale architectures in solution [25,26,27,28,29,30,31,32,33]. It is thus highly interesting to now exploit this concept of polyelectrolyte-organic counterion assembly to promote electrostatic self-assembly for the formation of functional nanoassemblies in solution.

Porphyrins and metalloporphyrins play a key role in several fundamental processes in life [34,35,36,37,38,39,40]. Extensive efforts have been undertaken to mimic efficient photoinduced electron-transfer processes which take place in photosynthesis and to create artificial systems. Among the variety of examined electron donors, porphyrins are very promising as they are the skeleton of chlorophyll and absorb intensively in the visible spectrum and exhibit high extinction coefficients [34,40,41,42,43,44,45,46]. Therefore, they have become very attractive for applications in various fields [47,48,49,50,51,52,53]. Water-soluble porphyrins are of particular interest due to their application in biological or medical fields [54]. The aggregation behavior of porphyrins has been studied extensively, and they have been combined with many-faceted molecules in supramolecular chemistry [55,56,57,58,59,60,61,62,63,64,65,66,67]. Porphyrins are often used for hydrogen production and for hydrogen storage which is an important challenge for the automotive industry [68,69,70,71,72,73,74,75,76]. Moreover, porphyrins can form interesting structures which are advantageous for several applications. Hasobe *et al.* reported the formation of hexagonal hollow nanotubes consisting of zinc *meso*-tetra(4-pyridyl) porphyrin which show a high power conversion efficiency (PCE) when filled with C_60_ or C_70_ [77].

Under acidic conditions the number of charges of free-base porphyrins can be tuned from four to six, resulting in porphyrin diacids, which also show interesting photophysical properties: They exhibit long-lived triplet states and their Q-bands appear in the near-IR-region. Therefore, a large fraction of the solar spectrum can be collected and hence porphyrin diacids represent potentially good photosensitizers [78,79,80,81]. In comparison to free-base and metalloporphyrins, however, much less attention has been paid to porphyrin diacids.

We recently found that porphyrin-polyelectrolyte assemblies with a tetravalent porphyrin show a substantially higher catalytic activity than pure porphyrin under neutral conditions [67].

**Scheme 1:**
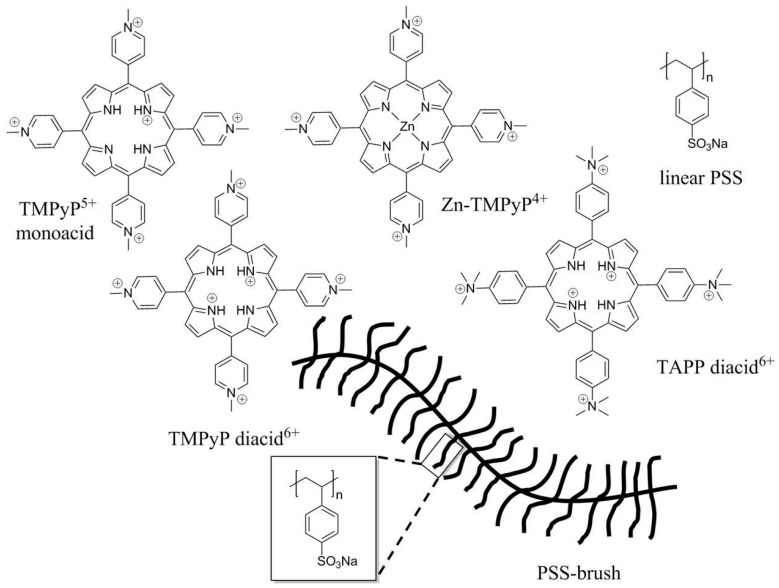
Building blocks used in this study: Cylindrical polystyrene sulfonate brushes or linear polystyrene sulfonate are combined with cationic porphyrins to build nanoscale assemblies in acidic aqueous solution.

**Scheme 2:**
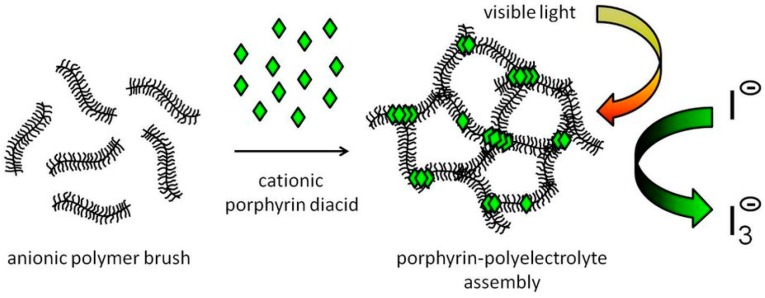
Formation of a supramolecular polyelectrolyte-porphyrin photocatalyst by electrostatic self-assembly.

Herein, for the first time, we investigate the catalytic activity of porphyrin-polymer assemblies under strongly acidic conditions where the free-base porphyrins carry two additional charges in the inner ring and in total are six-fold positively charged. We compare free-base porphyrin with the Zn-TMPyP as a metalloporphyrin. The building blocks used in this study are illustrated in Scheme 1. As macroion component, either an anionic cylindrical poly(styrene sulfonate) brush molecule (PSS brush) with about 12 nm diameter and 100 nm length or the corresponding anionic linear polystyrene sulfonate is used. These polyelectrolytes are combined with cationic *meso*-tetrakis(4-*N*-methylpyridinium) porphyrin (TMPyP), *meso*-tetrakis(4-(trimethylammonium) phenyl)-porphyrin (TAPP) or tetravalent *meso*-tetrakis-(*N*-methyl-4-pyridyl)-porphyrin-Zn(II) tetrachloride. The chosen model reaction for the investigation of the catalytic activity is the light-induced oxidation of iodide in aerobic conditions. The study shows that efficient supramolecular catalysts for photooxidation can be formed by electrostatic self-assembly in aqueous solution, as indicated in Scheme 2.

## 2. Materials and Methods

### 2.1. Chemicals

TAPP, TMPyP and potassium iodide were purchased from Sigma-Aldrich, Selmsdorf, Germany. Zn-TMPyP was purchased from TriPorTech, Selmsdorf, Germany. The cylindrical PSS brush was synthesized through polymerization of polystyrene macromonomers and subsequent sulfonation as described previously [82]. A polystyrene macromonomer was synthesized by anionic polymerization of styrene and subsequent end-functionalization by *p*-vinylbenzyl chloride. The macromonomer was characterized by MALDI-TOF giving *M*_w_ = 4450 g·mol^−1^ with *M*_w_/*M*_n_ = 1.06 from size exclusion chromatography (SEC) analysis (defining the later side chain length of the polymer brush being *P*_w,side chain_ = 43). Free radical homopolymerization of this macromonomer yielded a polystyrene brush with *M*_w_ = 2.13 × 10^6^ g·mol^−1^ and *M*_w_/*M*_n_ = 3.02 (SEC). To obtain lower polydispersities the polystyrene brush was fractionated by continuous polymer fractionation (CPF). A high molecular mass fraction with *M*_w_ = 4.12 × 10^6^ g·mol^−1^ and *M*_w_/*M*_n_ = 1.51 (weight average degree of polymerization of the total polymer *P*_w,total_ = 39,000 and of the main chain *P*_w,main chain_ = 900) was chosen. The polystyrene brush was then sulfonated with sulfuric acid/acetic anhydride in 1,2-dichloroethane at 50 °C. This results in a NaPSS brush with 100% sulfonation according to elemental analysis. Light-scattering analysis confirmed the polymeranalogous reaction to take place without degradation. Linear polystyrene was purchased from PSS standards with a molecular mass fraction with *M*_w_ = 666,000 g·mol^−1^ and a PDI < 1.2.

### 2.2. Sample Preparation

Samples were prepared by mixing aqueous solutions of the components and keeping them in darkness prior to further investigations. The irradiation was performed with a 300 W halogen lamp with a visible spectrum similar to daylight.

### 2.3. UV/Vis Spectroscopy

UV/Vis spectra were recorded using a SHIMADZU UV Spectrophotometer (UV-1800), Kyoto, Japan, with a slit width of 1 nm using quart cuvettes from Hellma with 1 and 10 cm path length. The spectral range covered 200 nm ≤ λ ≤ 800 nm.

### 2.4. Atomic Force Microscopy

Measurements were performed with a SolverPro AFM (NT-MDT Co., Moscow, Russia) equipped with a 50 μm scanner, an optical zoom and a damping activated table. The tip in tapping mode was a HA-NC noncontact mode cantilever with a resonance frequency of 130–180 kHz and a spring constant of 4.5 nm^−1^ (±20%) (also NT-MDT). For sample preparation, a droplet of 20 μL of the sample was added onto a freshly prepared mica surface and incubated for 5 min and dried in air. The TAPP-PSS brush sample l = 0.4 was spincoated at 3000 rpm.

### 2.5. Small Angle Neutron Scattering (SANS)

Measurements were performed at beamline KWS2 at the Jülich Centre for Neutron Science at the Heinz Maier-Leibnitz Zentrum (MLZ), Garching, Germany, and at the beamline D11 at Institut Laue Langevin (ILL), Grenoble, France. Data shown result from MLZ. Three configurations with neutron wavelengths λ = 4.55 Å and a sample-detector distance of *d* = 2 m, *d* = 8 m and *d* = 20 m were used. Data were corrected for empty cell scattering, electronic background, detector uniformity and analyzed after subtracting solvent scattering and incoherent background. Error bars lie within 1%–5% at low q and increase up to 15% at 0.9 nm^−1^ ≤ *q*.

## 3. Results

An important parameter for the investigation of the assemblies is the loading ratio, which is the molar ratio of porphyrin charges to polyelectrolyte charges:

(1) $$l_{\text{charge}}=\frac{c(\;{\text{–NR}}_{3}^{+},\text{Porphyrin})}{c(\;{\text{–SO}}_{3}^{-},\text{Polyelectrolyte})}$$

The structural investigation of the catalytically active aggregates takes place via atomic force microscopy (AFM) and small angle neutron scattering (SANS).

Under strong acidic conditions (pH < 2) the two inner nitrogen bases of a metal-free porphyrin ring become protonated leading to a hexavalent species in the case of cationic tetraphenylporphyrins accompanied by a strong colour change from red to green [83]. The valency increase from four to six is accompanied by an increase in symmetry from D_2H_ to D_4H_. Therefore, the planarity of the macrocycle of TMPyP and TAPP disappears and the metal-free porphyrins have the same symmetry as for example Zn-TMPyP. In the case of TMPyP, both, a pentavalent and a hexavalent porphyrin, can be received depending on pH. For the TMPyP monoacid pH 1.7 is sufficient whereas the diaicd is generated by dissolving the porphyrin directly in 1 M hydrochloric acid, *i.e.*, at pH = 0. Going from the tetravalent free-base porphyrin to the pentavalent TMPyP monoacid leads to a decrease of symmetry from D_2H_ to C_2V_. This one additional charge has a tremendous effect on the catalytic activity as will be discussed in the following.

### 3.1. Atomic Force Microscopy (AFM)

**Figure 1:**
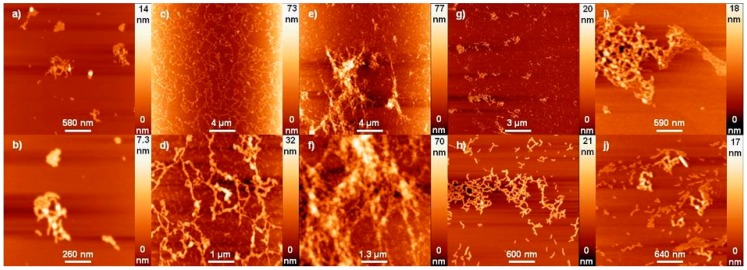
Atomic force microscopy (AFM) of porphyrin/PSS brush samples deposited onto a mica surface: (**a**,**b**) TAPP diacid-PSS brush sample with *l* = 0.4; (**c**,**d**) TMPyP diacid-PSS brush sample with *l* = 0.4; (**e**,**f**) TMPyP monoacid-PSS brush sample with *l* = 0.4; (**g**,**h**) TMPyP-PSS brush *l* = 0.5 in neutral solution; (**i**,**j**) Zn-TMPyP-PSS brush *l* = 0.4 in neutral solution.

In the AFM images shown in Figure 1, a loading ratio *l* = 0.4 was chosen for comparison, as this turned out to be the ratio with the highest catalytic activity under neutral conditions [31]. Structural differences are evident for the aggregates formed by the different porphyrin species with PSS brush. Figure 1a,b displays network-like structures formed by TAPP diacid and PSS brush, which were already found under neutral conditions [26,30]. Under acidic conditions these networks exhibit smaller dimensions in length (up to 645 nm) and even smaller dimensions in height (4 to 5.5 nm) as compared to former studies under neutral conditions that showed TAPP-PSS brush assemblies several μm in length and up to 15 nm in height [26].

To investigate the influence of each additional charge in the case of TMPyP systematically, the structure of TMPyP-PSS brush samples under neutral conditions was investigated (Figure 1g,h). Under neutral conditions, TMPyP-PSS brush forms well-defined, network-like structures which are 21 nm in height and exhibit also slightly larger meshes. In contrast, TMPyP monoacid (Figure 1e,f) forms rather undefined networks with several μm size which are up to 64 nm high. Hence, one additional charge causes a transition from well-defined network-like structures to rather undefined networks. Increasing the number of charges further so that the TMPyP diacid (Figure 1c,d) is present, again well-defined network-like structures result. They exhibit a height of 32 nm. Considering the PSS brush structures in the obtained aggregates more closely, one can see that the diameter of the PSS brush is up to twice as large in TMPyP diacid-PSS brush aggregates (Figure 1d) than under neutral conditions (Figure 1h). For the TAPP-PSS brush, one can see that there is nearly no difference in PSS brush width in the two considered pH regions. Furthermore, Figure 1i,j displays network-like structures for Zn-TMPyP and PSS brush under neutral conditions which are 17 nm in height and several hundreds of nm in size and which are therefore very similar to structures formed with TAPP and TMPyP under equivalent conditions (Figure 1g,h).

Several reasons could be responsible for the formation of well-defined or rather undefined structures with the different TMPyP species. The even or odd number of charges may be one reason for this because the PSS brush molecules can distribute less regularly with only one charge in the porphyrin ring. Increasing the number of charges from five to six, networks with TMPyP diacid (Figure 1d) (32 nm) are half as high as those with TMPyP monoacid (64 nm) and much more defined. Structural differences between TMPyP monoacid and TMPyP diacid can derive from their different symmetry (C_2V_ D_4H_). In addition, differences of the porphyrins TMPyP and TAPP can originate from the different ionic strengths. pK_a_ values for TAPP diacid and for TMPyP diacid are 3.6 and 1.4, respectively, indicating that TMPyP is the most acidic and the ionic strength is much larger in the TMPyP diacid sample than in the TAPP diacid sample. Usually, high ionic strength leads to a decrease in porphyrin-porphyrin charge repulsion due to screening and therefore the π-systems of two porphyrin macrocycles are more prone to interact intermolecularly. One might expect that the networks of TMPyP diacid-PSS brush exhibit tighter meshes due to the higher ionic charge, but it is in fact the opposite. The reason for this is not directly evident. High ionic strength was also investigated for TAPP diacid-PSS brush by dissolving both also directly in 1 M hydrochloric acid. The sample precipitates immediately and investigation with AFM was not possible. At higher ionic strength, screening of the electrostatic forces takes place leading to a stronger aggregation tendency. The structural investigation by AFM showed that TAPP diacid in combination with PSS brush also forms network-like structures similar to those under neutral conditions. In contrast, TMPyP diacid assembles into “huge” broader meshed networks and TMPyP monoacid makes larger undefined structures with PSS brush.

### 3.2. Structural Investigation by Small Angle Neutron Scattering (SANS)

**Figure 2:**
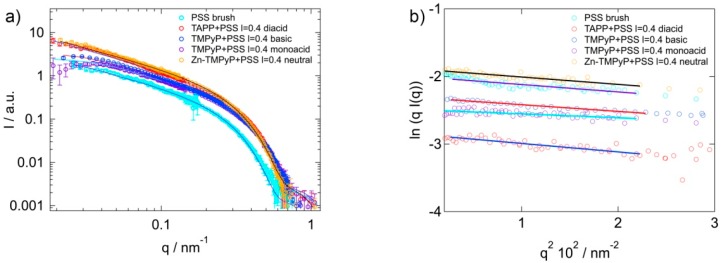
Small angle neutron scattering of PSS brush and different porphyrins: (**a**) scattering curves I(q); (**b**) cross-section Guinier plot. Straight lines reflect the corresponding fit.

To gain further insight into the structure and the shape of the aggregates in solution, small angle neutron scattering (SANS) measurements for each system were performed at polyelectrolyte concentrations of c (PSS brush) = 1 g·L^−1^, except for the TMPyP diacid sample where the concentration was c (PSS brush) = 0.05 g·L^−1^. Data are reported in Figure 2a. Scattering curves (Figure 2a) give evidence of the cylindrical shape of the PSS brush as the slope in a log/log representation for the intermediate *q*-range is −1.09, according to the scaling of the form factor *P*(q) with *q*^−1^ for long rods. From the first point or from the point where a plateau can be seen, the minimum length or approximate length of the cylinder is found via *l* = 2π/*q*_min_ which is approximately 250 nm. To gain more information, the curves have been analyzed by Guinier analysis (Figure 2b). The linearity of a cross-section Guinier plot confirms the cylinder shape. From the slope, the cross-section radius of gyration *R*_Gc_, can be obtained, which for the PSS brush is *R*_Gc_ = 4.8 nm. Assuming a homogeneous structure, this *R*_Gc_ can be converted into a cross-section radius and consequently into a diameter, which is 13.8 nm, in good agreement with former studies [26,30]. To see how the porphyrin influences the PSS brush in the aggregates, samples with the same loading ratio *l* were investigated for each porphyrin.

In each case, cylindrical nanoassemblies were found. Table 1 shows the cylinder lengths, *R*_Gc_ and radii. On the basis of this first analysis, the experimental curves have been fitted according to structural models to obtain the particle shape and dimensions. Results are given in Figure 2a (solid lines) and Table 1. It can be seen that all the determined radii are smaller than the one of the PSS brush alone, while lying all in the same range. TMPyP diacid-PSS brush differs, as with 12 nm the radius is nearly twice as large as that of the PSS brush. The result fits well with the observations during sample preparation as precipitation occurs immediately for polyelectrolyte concentrations of *c* (PSS brush) = 1 g·L^−1^, which is why a distinct smaller concentration has to be used.

Hence, SANS results showed that cylindrical aggregates are formed, which is consistent with AFM where individual strands of the networks exhibit a cylindrical shape. The lengths of these cylinders range from 93 to 2700 nm. Differences in length are also evident from the AFM images: for the TMPyP diacid-PSS brush sample, large network-like structures were found, the single-strand dimensions of which are given in comparison are the longest and widest. The largest radius for the cylinders formed by TMPyP diacid and PSS brush is in good agreement to the corresponding AFM. An exception is the TMPyP monoacid, where structures found in AFM are quite undefined. All the diameters are smaller than that of TMPyP diacid and also the determined lengths are all distinctly smaller. Hence, overall SANS and AFM results are in very good agreement.

The observation that, with the exception of TMPyP daicid, the radii are all smaller than those of PSS brush in a broader view agrees very well with observations from former studies, where SANS measurements under neutral conditions with and without salt were performed [62,66]. It was observed that PSS brush had the largest radius followed by TAPP-PSS brush and TMPyP-PSS brush. Thus the shrinkage herein can derive from the larger number of charges of the porphyrin, which causes the porphyrin to enter more into the inside of the PSS brush, as the PSS brush has more power to bind it due to the additional two charges and therefore smaller diameters result. Former studies pointed out that side-chain interconnections seem to be responsible for the smaller diameters of the porphyrin-PSS brush assemblies under neutral conditions, [30] which also is the case for TAPP diacid and TMPyP monoacid. The behavior of TMPyP diacid, in contrast, appears to be different and is not understood yet.

### 3.3. Spectroscopic Investigation

**Figure 3:**
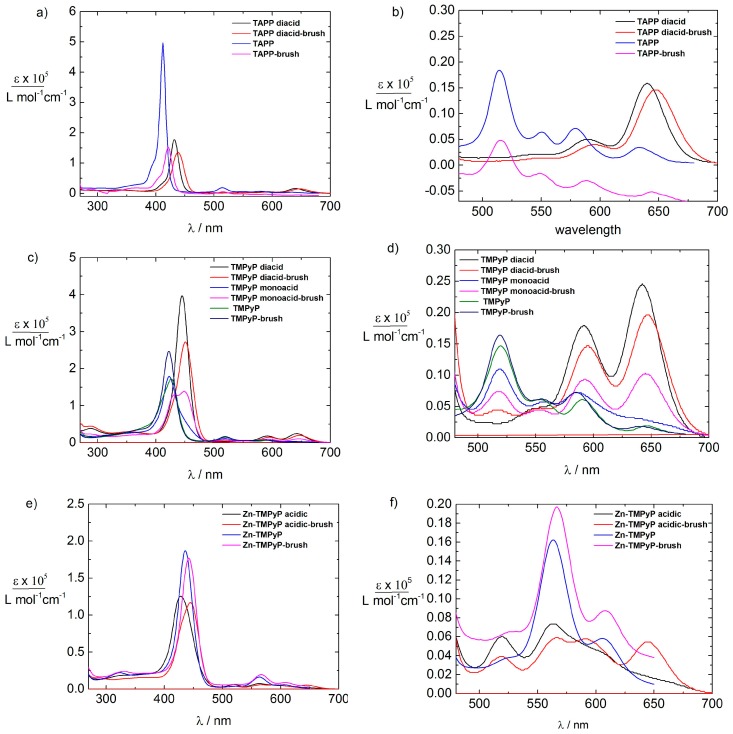
Spectral changes of porphyrin-PSS brush aggregates upon changing the pH from neutral to highly acidic: (**a**) TAPP-sample with PSS brush *l* = 0.4; (**b**) Zoom of the Q bands of (**a**); (**c**) TMPyP-sample with PSS brush *l* = 0.1 for the diacid and monoacid, *l* = 0.4 under neutral conditions; (**d**) Zoom of the Q bands of (**c**); and (**e**) Zn-TMPyP-sample with PSS brush *l* = 0.4; (**f**) Zoom of the Q bands of (**e**).

The difference in valency becomes evident spectrochemically as can be seen in Figure 3. Here, samples with and without polyelectrolyte under neutral and acidic conditions are investigated for the three different porphyrins. Figure 3a–c exhibit the complete spectrum, whereas Figure 3d–f focus on the enlarged Q-bands. Under neutral conditions, the Soret band of pure TAPP (Figure 3a) is red-shifted for TAPP-PSS brush aggregates with *l* = 0.4, indicating the formation of J-aggregates and a head-to-tail interaction of the transition dipole moments. Changing the pH from neutral to acidic, the TAPP Soret band undergoes a bathochromic shift indicating the formation of J-aggregates. This bathochromic shift with 9 nm under neutral conditions is slightly larger than under acidic conditions with 6 nm indicating larger interactions between the TAPP and the PSS brush or the formation of larger J-aggregates under neutral conditions. This pH change is, as already mentioned, accompanied by an increase of symmetry, which again is accompanied by a reduction of the number of Q-bands from four to two. The presence of PSS brush leads to a small further red-shift of the TAPP diacid Soret band (Figure 3b). The Q-bands undergo no spectral shift upon combination with the polyelectrolyte under neutral conditions, while this is different under acidic conditions. Here, a slight bathochromic shift of the Q-bands in the presence of PSS brush can be seen. Spectral data from Figure 3 are summarized in Table 2. In the case of TMPyP (Figure 3c), no band shifts of the Soret band from the neutral TMPyP to TMPyP monoacid can be observed. The presence of PSS brush in neutral conditions leads to a small change of the Soret band characteristics but not to a band shift. For the TMPyP diacid, a clear bathochromic shift of the Soret band can be observed indicating the formation of J-aggregates and a head-to-tail interaction of the transition dipole moments as for TAPP diacid, which in the presence of PSS brush is slightly more expressed. For TMPyP monoacid, the presence of PSS brush leads to a red-shift of the Soret band and for the illustrated charge ratio to a band splitting, indicating that more than one dominant species exists. In addition, in the Q-region, some spectral shifts occur (Figure 3d). For the neutral TMPyP samples, a slight red-shift of the Q-bands with polyelectrolyte addition can be seen. This is also the case for the TMPyP diacid. For Zn-TMPyP (Figure 3e) less spectral changes are expected, because the metal center prevents the increase of charge and also symmetry changes are not expected. Thus, only for pure Zn-TMPyP Soret band can a slight blue shift by changing the milieu from neutral to acidic be observed, thereby indicating the formation of H-aggregates and a face-to-face interaction. However, the corresponding Q-bands show shifts (Figure 3f).

For the pure Zn-TMPyP in solution, the first Q-band rises at 519 nm, the second at 563 nm and, additionally, a weak shoulder at higher wavelengths is present. In the presence of the PSS brush under acidic conditions, four Q-bands can be seen, which are almost at the same wavelengths as TMPyP under neutral conditions, indicating that Zn-TMPyP becomes demetallated under such acidic conditions.

The spectroscopic investigation therefore showed that the structure formation of porphyrin and polyelectrolyte always leads to a red-shift indicating the formation of J-aggregates. For the TMPyP monoacid, additionally a band splitting can be observed indicating the presence of more than one species, which fits very well to the rather undefined structures in AFM.

### 3.4. Catalysis

Recently we found that at pH = 7 porphyrin-polyelectrolyte assemblies catalyze the light-induced oxidation of iodide in aqueous solution more effectively than the unassociated porphyrin [67]. Here, we performed a study of the catalysis of different porphyrins under strongly acidic conditions. The free-base porphyrins are two-fold protonated, resulting in two additional charges, *i.e.*, hexavalent porphyrins. Due to the fact that electrostatic interactions between the positively charged porphyrins and the negatively charged polyelectrolytes are responsible for structure formation and the structure formation itself under neutral conditions has caused a higher catalytic performance, two additional charges are expected to have a substantial influence on the catalytic activity. Therefore, porphyrin diacid-polyelectrolyte assemblies are promising for catalysis especially under strong acidic conditions where a variety of systems cannot be used. For the main part of the catalysis study, three different porphyrins, TAPP, TMPyP and Zn-TMPyP, are each combined with a cylindrical PSS brush and the catalytic activity of these aggregates was compared with the one of porphyrin only in acidic solution. In addition, measurements were also done with linear PSS to identify the role of the polyelectrolyte architecture.

**Figure 4:**
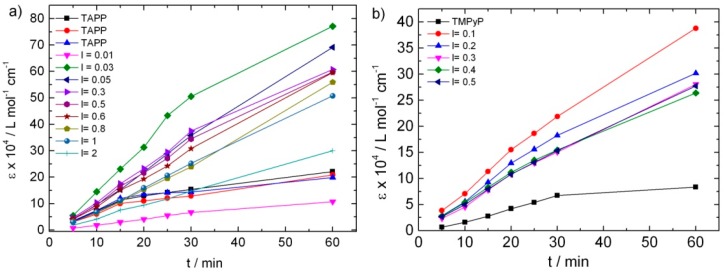

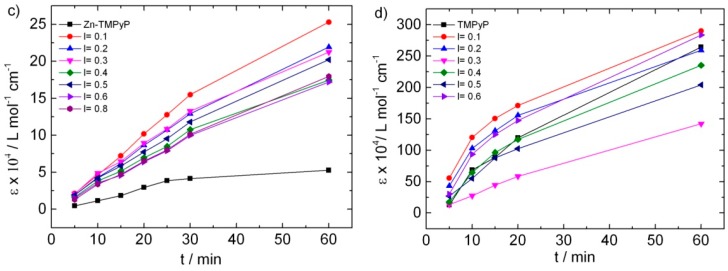
Kinetics of tri-iodide formation: extinction coefficient ε at λ = 353 nm *versus* irradiation time for samples in acidic iodide solution: (**a**) TAPP diacid-PSS brush; (**b**) TMPyP monoacid-PSS brush; (**c**) Zn-TMPyP-PSS brush; (**d**) TMPyP diacid-PSS brush.

The chosen model reaction is the light-induced oxidation of iodide into triiodide, because I^−^/I_3_^−^ is used, for example, in solar cell applications. The generation of triiodide can be monitored through two characteristic absorption bands at 287 and 353 nm in the UV/Vis spectrum. The development of the triiodide absorption as a function of time for the different systems is plotted in Figure 4. Triiodide concentrations are summarized in Table 3. The system of TAPP diacid and PSS brush is exemplarily chosen and the influence of the loading ratio *l* on the catalytic activity was investigated over a large *l* regime 0.01 ≤ *l* ≤ 2. As can be seen from Figure 4a, the catalytic activity of TAPP diacid-PSS brush assemblies increases successively with an increasing amount of polyelectrolyte: that is, the concentration of triiodide increases from *l* = 2 successive up to *l* = 0.03 where the highest catalytic activity can be observed. Further increase of polyelectrolyte leads to a smaller catalytic activity, which shows that *l* = 0.03 is the optimum loading ratio which is necessary for an improvement of the catalytic activity. At *l* = 0.01, the amount of TAPP is too small to allow for building sufficient aggregates. Between 0.3 ≤ *l* ≤ 0.6, no differences can be seen. Similar observations can be made for the TMPyP monoacid-PSS brush system, where the considered regime was 0.1 ≤ *l* ≤ 0.5. As can be seen in Figure 4b, a distinct difference in the catalytic activity with and without polyelectrolyte can be observed. The highest catalytic activity was found for *l* = 0.1. For the Zn-TMPyP-PSS brush system, the results of which are illustrated in Figure 4c, samples with 0.1 ≤ *l* ≤ 0.8 were investigated. Similar to the results for the TMPyP monoacid-PSS brush system, a distinct increase in catalytic activity through the polyelectrolyte can be seen.

Hence, the activity increases with increasing amount of polyelectrolyte so that for *l* = 0.1 the highest amount of triiodide was found. For the TMPyP diacid-PSS brush system, the observations are different as can be seen in Figure 4d. The pure TMPyP diacid solution is the catalytically most active one of the investigated porphyrin only solutions. With polyelectrolyte, the catalytic activity is the same as without polyelectrolyte. Among the considered loading ratios, *l* = 0.1 shows the highest catalytic activity, but the activity does not continuously increase with increasing amount of polyelectrolyte. Among the considered systems, TMPyP diacid-PSS brush assemblies with c (I_3_^−^) = 4.2 × 10^−4^ mol·L^−1^ generate the highest concentration of triiodide followed by TAPP diacid with c (I_3_^−^) = 1.5 × 10^4^ mol·L^−1^, Zn-TMPyP with c (I_3_^−^) = 8.7 × 10^−5^ mol·L^−1^ and TMPyP monoacid with c (I_3_^−^) = 7.8 × 10^−5^ mol·L^−1^, as can be seen in Table 3.

These observations are also evident from the corresponding turnover numbers (TON) and the turnover frequency (TOF), which are summarized in Table 4. TON describes the amount of triiodide which can be generated with the chosen porphyrin concentration. TOF is the turnover per time. The catalytic activity of TMPyP diacid-PSS brush assemblies is evident with a TON of 109 and a TOF of 1.82, which are higher than those of pure TMPyP diacid and essentially larger than those of the other systems. Thus, the catalytic activity of TAPP diacid, TMPyP monoacid and Zn-TMPyP can be obviously enhanced with regard to triiodide generation in the presence of polyelectrolyte.

From the results it can be seen that one additional charge in the case of TMPyP enhances the catalytic activity tremendously. The effect of the porphyrin diacids is much more significant than with the porphyrin under neutral pH conditions [31]. Under acidic conditions, the triiodide generation is up to 13.6 times larger than in the pH 7 case in the TAPP diacid-PSS brush and up to 4.1 times larger in the case of TMPyP monoacid-PSS brush, and finally up to 22.1 times larger in the case of TMPyP diacid-PSS brush.

**Figure 5:**
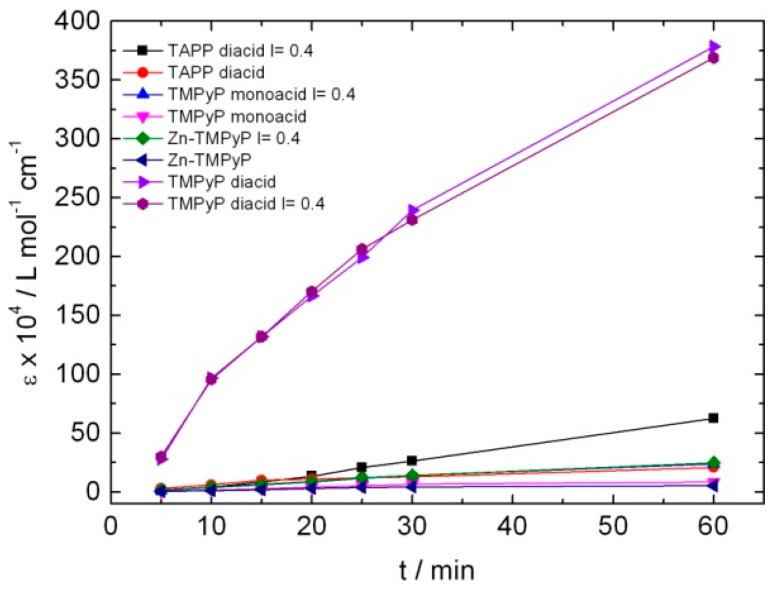
Kinetics of triiodide formation: extinction coefficient ε at λ = 353 nm *versus* irradiation time for porphyrin-linear PSS in acidic aqueous iodide solution.

**Figure 6:**
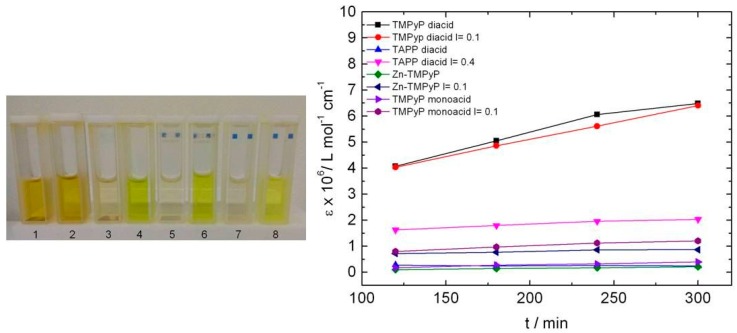
(**Left**) Image of samples after 2 h of irradiation with visible light. The increased catalytic activity can be seen by eye. Samples with polyelectrolyte (2, 4, 6, 8) are intensively yellow-colored. 1, 2: TMPyP diacid; 3, 4: TAPP diacid; 5, 6: Zn-TMPyP; 7, 8: TMPyP monoacid; (**Right**) kinetics of triiodide formation: extinction coefficient ε at λ = 353 nm *versus* irradiation time (5 h) for different porphyrin-PSS brush assemblies in acidic aqueous iodide solution.

To identify if a certain polyelectrolyte architecture is necessary to increase the catalytic activity, measurements with linear PSS as polyelectrolyte at one chosen loading ratio *l* = 0.4 were performed. As shown in Figure 5, also with the linear polyelectrolyte, the catalytic activity becomes enhanced whereas in the case of TMPyP diacid, the catalytic activity without linear PSS is slightly higher than that with linear PSS. The concentrations of generated triioide are slightly higher for the TMPyP diacid with linear PSS as with the PSS brush, while the other porphyrins lie in the same range as with PSS brush, as given in Table 5. This is in contrast to neutral conditions. From Figure 5 and Table 5, it is evident that most triiodide is generated with the TMPyP diacid system, which is consistent with the results of the PSS brush. For selected samples, the influence of long-time irradiation on the catalytic performance of porphyrin diacid-PSS brush aggregates was investigated. Samples were irradiated up to five hours. A more extended irradiation interval was not possible for the TMPyP diacid samples due to absorption limits reached at the chosen concentration. Again, the extinction coefficients are plotted versus the irradiation time in Figure 6. It becomes evident that the further increase of the catalytic activity due to the four-hour longer irradiation is not that significant for the majority of the porphyrin-polyelectrolyte samples. Only for the TMPyP diacid system can a clear enhancement of the catalytic activity be observed. Consistent with the results for one-hour irradiation, the highest concentration of generated triiodide is found for TMPyP diacid-PSS brush aggregates, as summarized in Table 5. Yet, still no increase of the iodide concentration due to the polyelectrolyte can be seen. The activity of both samples is nearly the same as after one-hour irradiation. For the remaining three porphyrin-PSS brush systems, a clear increase of the catalytic activity caused by the polyelectrolyte is evident. Again, the observations can be underlined with the corresponding TON and TOF, which are summarized in Table 6. The maximum increase with a 7.5-times higher concentration of generated triiodide was found for TAPP diacid-PSS brush.

The increase of the catalytic activity can additionally be seen from the color of the investigated samples in Figure 6. Samples with porphyrin and polyelectrolyte evidently are more intensively yellow-colored.

The reusability of the porphyrin diacid-PSS brush-assemblies was also investigated. For this, a sample of TAPP diaicd-PSS brush and a TAPP diacid solution, which were already irradiated and used as catalyst, were irradiated on the next day again. The measurements showed that TAPP diacid-PSS brush assemblies are still catalytically active.

## 4. Discussion

**Scheme 3:**
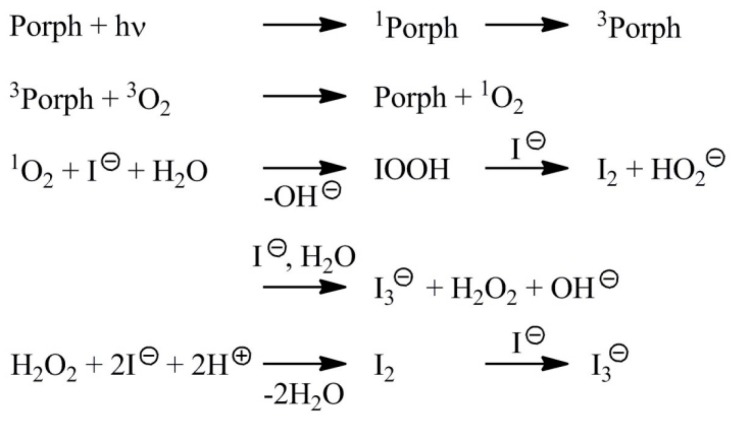
Reaction scheme of the investigated model reaction.

Possible origins for the difference in catalytic activity are different lifetimes of the excited triplet states, the symmetry of the porphyrins as well as the electronic structure of the porphyrins. As shown in Scheme 3, the generation of triiodide from iodide occurs via irradiation of the porphyrin as a photosensitizer and its excited triplet state. Thereby, ^1^O_2_ is generated which oxidizes iodide into triiodide. Table 7 summarizes the excited triplet state lifetimes for the porphyrins used. From this, it may be assumed that Zn-TMPyP should exhibit the highest catalytic activity under neutral conditions due to the largest lifetime and that TAPP should be more catalytically active than TMPyP, both under neutral and acidic conditions.

Results above show the opposite. Therefore, effects other than the lifetimes of the excited triplet states evidently are more significant for the difference in catalytic activity. Generally, molecular symmetry can also play a role for the different catalytic behavior, but TMPyP and TAPP exhibit the same symmetry under neutral and acidic conditions, so that the molecular symmetry also turns out not to be the reason for the difference in catalytic activity.

**Figure 7:**
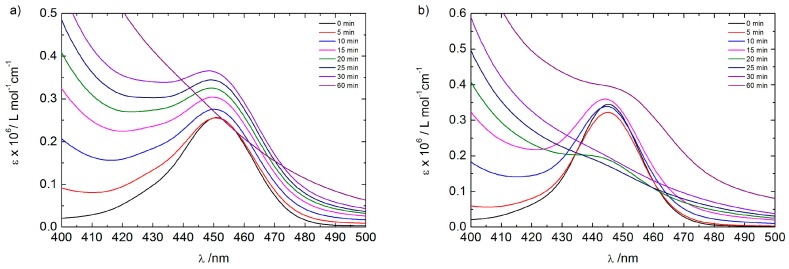

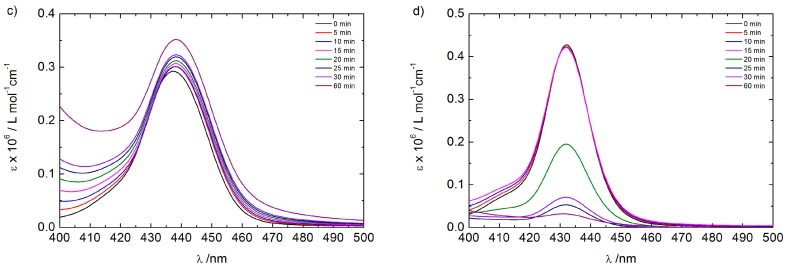
Change of the porphyrin Soret band during irradiation; (**a**) TMPyP diacid + PSS brush, *l* = 0.1; (**b**) TMPyP diacid; (**c**) TAPP diacid + PSS brush, *l* = 0.4; (**d**) TAPP diacid.

A likely cause contributing to the enhancement of the catalytic activity is the prevention of uncontrolled aggregation, as previously observed under neutral conditions. Therefore, for porphyrin diacid-PSS brush systems, the change of the Soret band during irradiation was considered in detail (Figure 7). The behavior is quite complex. With polyelectrolyte, the intensity of the Soret band increases with increasing irradiation time until its disappearance after 60 min of irradiation. Without polyelectrolyte, one can first see a decrease of the Soret band and afterwards an increase and disappearance of the Soret band already after 20 min of irradiation. This observation indicates that TMPyP diacid-PSS brush assemblies are destroyed with increasing irradiation time, and more free TMPyP diacid becomes present in the solution. Different observations can be made in the case of TAPP diacid. With polyelectrolyte, only a slight decrease of the Soret band can be observed, whereas without polyelectrolyte, the Soret band decreases, indicating undefined aggregation, which is prevented by the PSS brush in the assembly system. The same result can be seen for TMPyP monoacid and Zn-TMPyP. This observation of the Soret band behavior fits well with the long-term photocatalytic activity results. There, the amount of generated triiodide for the TMPyP diacid sample and for the TMPyP diacid-PSS brush sample in relation to the short-term experiment did not increase tremendously. With the results from Figure 7 it becomes evident that this is because TMPyP diacid-PSS brush assemblies are not stable enough under the investigated conditions, so that after one-hour irradiation, aggregates no longer exist and therefore the catalytic activity of the samples with and without polyelectrolyte is the same. In the case of TAPP diacid, the aggregates are more stable and are still present after one-hour irradiation and therefore a distinctly larger increase of the triiodide concentration can be observed in the long-term studies. In summary, the prevention of aggregation through the polyelectrolyte is likely the reason for the enhanced catalytic activity of TAPP diacid, while for TMPyP diacid, this appears not to be the case.

Differences between the TMPyP and TAPP system can derive from their electronic structures. Wang investigated the interactions of porphyrin-borate complexes and he also applied density functional theory [86]. For TMPyP, he observed a redshift of the Soret band when complexed with borate resulting from the reduced energy levels of the TMPyP in complexes. The methylpyridinium groups with their four positive charges exhibit an electron-withdrawing character and have both an inductive and a mesomeric effect. The inductive effect appears to have no influence on the energy of the π-orbitals. It proceeds via the σ-bonds of the substituents towards the porphyrin σ-system. On the contrary, the mesomeric effect reflects proceeds via the porphyrin π-system. For resonance interaction, the substituents need to rotate towards a coplanar configuration. Therefore, the dihedral angles between the methylpyridinium groups and the pyrrole rings for the TMPyP monomer were considered: all are 67.30° and become smaller when TMPyP is complexed. This means that TMPyP exhibits a higher planarity and becomes flattened in porphyrin-borate complexes [86]. TAPP, conversely, has localized charges on the substituents, which cannot be delocalized onto the porphyrin π-system so effectively. Cho investigated the electronic perturbation of *meso*-substituted free-base porphyrins [87]. P-aminophenyl acts as an electron-donating group and additionally participates in the extension of the π-conjugation of the HOMO of the porphyrin unit. *P*-aminophenyl-sibstituted porphyrins should have characteristic properties resulting from the unique MO interactions of the porphyrin and the *p*-aminophenyl substituents. It was concluded that the *p*-aminophenyl substituent can efficiently perturb the π-electronic system of the porphyrin unit. Due to the fact that the dihedral angle is important for efficient interunit interactions, Cho also investigated the dihedral angle, revealing an angle between the porphyrin and *p*-aminophenyl, pentafluorophenyl or phenyl substituents that is approximately 70°. The *p*-aminophenyl substituent should be the energetically and geometrically most effective unit for interunit interactions with the porphyrin. The LUMOs of free-base porphyrins are localized on the porphyrin unit; however, electron delocalization through *p*-aminophenyl substituents has its origin in an intramolecular charge transfer character in the excited states. P-aminophenyl substituents with non-orthogonal geometry lead to efficient electron delocalization. This is supported by a large electron density on the *p*-aminophenyl substituent in the HOMO [87]. Thus, these are significant differences in the electronic structure of TMPyP and TAPP that can contribute to a different behavior in photocatalysis. With this understanding, the concept presented may be extended towards versatile and tunable photocatalytic structures that can be designed by electrostatic self-assembly, in particular with regard to solar energy conversion.

## 5. Conclusions

We have shown that electrostatic self-assembly of highly charged porphrins with polyelectrolytes in aqueous solution can yield a variety of nanostructures with significantly improved properties for photocatalysis. The study revealed an enhancement of the photocatalytic activity of different porphyrin diacids through assembly with the poly(styrene sulfonate) (PSS) brush. The diacids of *meso*-tetrakis(4-(trimethylammonium) phenyl)-porphyrin (TAPP) and *meso*-tetrakis(4-*N*-methylpyridinium) porphyrin (TMPyP) have been investigated as well as TMPyP monoacid and Zn-TMPyP. The results showed in the case of TMPyP that the more charges the porphyrin exhibits, the higher the generated amount of triiodide. TMPyP diacid-PSS brush assemblies generate up to 22 times more triiodide than TMPyP-PSS brush assemblies under neutral conditions and otherwise same conditions. The amount of polyelectrolyte also influences the catalytic activity of porphyrin-PSS brush assemblies: A maximum of catalytic activity enhancement was found for *l* = 0.03. Atomic force microscopy (AFM) revealed that porphyrin diacids assemble with PSS brush into larger networks with different density of meshes, which can be due to symmetry changes and a difference in ionic strength, while small angle neutron scattering (SANS) confirmed the cylindrical shape of the network moieties. The difference in catalytic activity was related to difference in electronic structure of the porphyrin and porphyrin-porphyrin interaction, while lifetimes and molecular geometry turned out to not be directly connected with the catalytic activity. Hence, electrostatic self-assembly of polyelectrolytes with multivalent functional counterions leads to functional nanostructures with tunable structure and activity. The advantage of the concept presented is a simple toolbox principle based on ionic interactions, which opens the route to a wide variety of tunable self-assembled catalysts formed with polyelectrolytes.

## Figures and Tables

**Table 1:** Cylinder lengths, cross-section radii and radii from SANS data.

Sample	*R*_GC_^1^ (nm)	Diameter ^1^ (nm)	Model	Length (nm)	Radius (nm)
PSSbrush neutral	4.8	13.8	Cylinder	140 ± 10	5.9 ± 0.1
TMpyP + PSSbrush	4	11.4	Cylinder	100 ± 10	5.1 ± 0.1
TMPyPmonoacid + PSSbrush *l* = 0.4	4.0	11.4	Cylinder	93 ± 3	4.8 ± 0.1
TMPyP diacid + PSS brush *l* = 0.4	–	–	Cylinder	2,700 *	12 ± 0.1
Zn-TMPyP + PSS brush *l* = 0.4	4.6	13	Cylinder	200 *	5.8 ± 0.1
TAPP diacid + PSSbrush *l* = 0.4	4.3	12.3	Cylinder	100 *	5.2 ± 0.1

^1^ Determined via Guinier approximation and modeling; * Length taken from AFM.

**Table 2:** Spectroscopic data for porphyrin-PSS brush assemblies in different media.

System	Soret-Band (nm)	Qy(1,0) (nm)	Qy(0,0) (nm)	Qx(1,0) (nm)	Qx(0,0) (nm)
TAPP diacid	432	589	640	–	–
TAPP diacid *l* = 0.4	438	594	647	–	–
TAPP	412	514	549	579	634
TAPP *l* = 0.4	421	516	549	588	644
TMPyP diacid	445	591	642	–	–
TMPyp diacid *l* = 0.1	450	595	647	–	–
TMPyp monoacid	423	518	558	586	
TMPyP monoacid *l* = 0.4	430/447	518	554	592	644
TMPyp	422	519	555	584	640
TMPyP *l* = 0.4	424	520	555	591	646
Zn-TMPyP acidic	428	519	563	–	–
Zn-TMPyP acidic *l* = 0.4	445	518	566/591/645	–	–
Zn-TMPyP	436	562	606	–	–
Zn-TMPyP *l* = 0.4	442	566	608	–	–

**Table 3:** Generated concentrations of triiodide (mol/L).

Porphyrin species	With PE	Without PE	Activity increase with PE
TMPyP diacid	4.2 × 10^4^	4.2 × 10^4^	unchanged
TAPP diacid	1.5 × 10^4^	5.9 × 10^5^	2.5×
Zn-TMPyP	8.7 × 10^5^	6.1 × 10^5^	1.3×
TMPyP monoacid	7.8 × 10^5^	2.3 × 10^5^	3.4×

**Table 4:** Corresponding TON/TOF of investigated porphyrin-polyelectrolyte assemblies.

Porphyrin species	TON		TOF/min^−1^	
	With PE	Without PE	With PE	Without PE
TMPyP diacid	109	104	1.82	1.73
TAPP diacid	20	6	0.66	0.1
TAPPneutral	2	–	0.03	–
Zn-TMPyP	8	47	0.14	0.8
TMPyP monoacid	10	3	0.14	0.04
TMPyP neutral	4	2	0.06	0.04

**Table 5:** Generated concentrations of triiodide (mol/L) after 5 h of irradiation.

Porphyrin species	With PE	Without PE	Increased activity with PE
TMPyP diacid	9.42 × 10^4^	1.00 × 10^3^	slightly less catalytically active
TAPP diacid	5.26 × 10^4^	7.01 × 10^5^	7.5×
Zn-TMPyP	2.59 × 10^4^	7.73 × 10^5^	3.6×
TMPyP monoacid	2.01 × 10^4^	7.35 × 10^5^	2.7×

**Table 6:** TON/TOF for the long-term studies of porphyrin-polyelectrolyte assemblies.

Porphyrin species	TON		TOF/min^−1^	
	With PE	Without PE	With PE	Without PE
TMPyP diacid	243	246	0.81	0.82
TAPP diacid	78	10	0.25	0.03
Zn-TMPyP	33	9	0.11	0.03
TMPyP monoacid	46	16	0.15	0.05

**Table 7:** Lifetimes of the excited triplet states of the different porphyrins under different conditions.

Porphyrin	pH	Φ_Triplet_	*t*_T_
TMPyP	7	0.92 [64]	0.17 ms [84]
TAPP	7	0.8 [65]	0.54 ms [85]
Zn-TMPyP	7	0.9 [64]	2 ms [84]
TMPyP diacid	acidic	–	90 μs [76]
TAPP diacid	acidic	–	268 μs [76]
Zn-TMPyP	acidic	–	–
